# Effect of the incubation time on blood culture results and bacterial pathogens causing bloodstream infections among children attending Sekou Toure Regional Referral Hospital in Mwanza, Tanzania

**DOI:** 10.1099/acmi.0.000942.v3

**Published:** 2025-03-19

**Authors:** James Thomas, Albert Wasira, Darus Maarafu, Faustin Igogo, Eunice Emmanuel, Roza Ernest, Martha F. Mushi, Stephen E. Mshana

**Affiliations:** 1 Weil Bugando School of Medicine, Catholic University of Health and Allied Sciences, P.O. Box 1464, Mwanza, Tanzania; 2 Medical Laboratory Science, Weil Bugando School of Medicine, Catholic University of Health and Allied Sciences, P.O. Box 1464, Mwanza, Tanzania; 3 Sekou Toure Regional Referral Hospital, P.O. Box 132, Mwanza, Tanzania; 4 Department of Microbiology and Immunology, Weil Bugando School of Medicine, Catholic University of Health and Allied Sciences, P.O. Box 1464, Mwanza, Tanzania

**Keywords:** antimicrobial resistance, blood culture, bloodstream infection, children, incubation time

## Abstract

**Background.** A one hour delay in initiating appropriate antimicrobial treatment increases the mortality rate of patients with bloodstream infections by 2%. This highlights the risk associated with manual blood culture methods, as they tend to have long turnaround time, with an initial incubation period of 18–24 h, leading to delays in obtaining diagnostic results. This study examined the impact of incubation time on blood culture results and analysed the patterns of the pathogens causing bloodstream infections (BSIs) among children attending Sekou Toure Regional Referral Hospital (SRRH), Mwanza, Tanzania

**Methodology.** A hospital-based, descriptive cross-sectional study was conducted at SRRH from May to July 2024. The conventional blood culture method, using in-house prepared brain heart infusion broth with slight modifications on the initial time of the blind subculture (at 8, 24 and 120 h) was done to isolate the pathogens causing BSIs. Descriptive data analysis was performed using STATA software version 15.

**Results.** The study enrolled 302 children with clinical diagnosis of BSIs. Of these, 160 (53%) were male, with a median age of 6 years interquartile range [IQR] 1–7 years. Fever was the predominant clinical sign reported in 259 (85.8%) children. Microbiologically confirmed BSIs were detected in 90 (29.8%) children. Among them, 51.1% (46/90) were detected through blind subculture after 8 h of initial incubation. An additional 31 (34.4%) and 13 (14.4%) were detected after 24 h and 120 h of incubation, respectively. The most frequently isolated pathogens were *Klebsiella pneumoniae* (25.6%, 23/90) and *Staphylococcus aureus* (24.4%, 22/90). Gram-negative bacteria (GNB) formed the majority (71.1%, 64/90) of the isolated pathogens, with 62.5% (40/64) showing resistance to third-generation cephalosporin (3GC). Additionally, 45.5% (10/22) of the *S. aureus* strains were methicillin-resistant *S. aureus*.

**Conclusion.** Blind subculture after 8 h of initial incubation correctly detected more than half of the children with microbiologically confirmed BSIs. Incorporating blind subculture on MacConkey agar supplemented with 2 µg ml^−1^ cefotaxime (MCA-C) after 8 h of incubation resulted in the correct treatment of half of the children with BSIs caused by GNB within 24 h. In areas with high prevalence of 3GC resistance, blind subculture within 8 h should include MCA-C for appropriate treatment within 24 h.

## Data Availability

All data involved in this study are included in the manuscript.

## Introduction

Bloodstream infection (BSI) is the leading cause of sepsis and the most pronounced cause of paediatric mortality worldwide [1]. Globally, 20.3 million incident cases of sepsis are recorded, which contribute to 2.9 million mortality annually in the paediatric population [2]. In low- and middle-income countries, the prevalence of sepsis is estimated to be two times higher than in high-income countries [3]. Antimicrobial resistance (AMR) among Gram-negative bacteria (GNB) causing BSI is reported to be high, with 60–90% of these isolates exhibiting extended-spectrum beta-lactamase (ESBL) phenotype [4]. The alarmingly high prevalence of ESBL-producing GNB causing BSIs highlights the urgent need for reliable methods to promptly detect these pathogens and initiate appropriate treatment. This is particularly critical, as ampicillin and gentamicin remain the first-line treatment for sepsis in children and neonates [5].

In Tanzania, the prevalence of sepsis in children is estimated to be 20%, with an attributable mortality of 20–50%, mainly among admitted patients [6,8]. At the Bugando Medical Centre (BMC), GNB were documented as the most common pathogen causing neonatal sepsis, accounting for 61.1% of cases with *Klebsiella pneumoniae* and *Escherichia coli* being predominant pathogens [9]. A subsequent study conducted 8 years later reported GNB as leading pathogens, accounting for 75% of cases [6]. In addition, these two studies reported significantly high mortality among children infected with extended spectrum beta-lactamase-producing *Enterobacterales,* mainly *K. pneumoniae* and *E. coli* [6,9]. Meanwhile, the report from the World Health Organization (WHO) indicated that neonatal mortality associated with sepsis can be reduced from 2.5 million to ~400 000 deaths annually through improved timely diagnosis and appropriate clinical management [2]. This highlights the need for early diagnosis and timely initiation of appropriate antibiotics in order to improve the outcomes of paediatric patients with BSI.

The routine conventional blood culture done in most of the health facilities in developing countries involves a blind subculture within 18–24 h post-incubation, with pathogen identification and susceptibility testing requiring an additional 24–36 h [8]. Given the fact that the majority of the pathogens causing BSIs in these settings are GNB with more than 50% of them being resistant to third-generation cephalosporins (3GCs) [6,8, 9], this study investigated the yield and the detection of GNB resistant to 3GC by incorporating blind subculture at 8 h post-initial incubation on plain MacConkey agar and MacConkey supplemented with 2 µg ml^−1^ cefotaxime (MCA-C), respectively. The overall aim of subculture in MCA-C was to shorten the time of detecting GNB resistant to 3GC.

## Methodology

### Study design and study period

This was a hospital-based, descriptive cross-sectional study that was conducted from 18 May to 31 July 2024.

### Study area

The study was conducted at Sekou Toure Regional Referral Hospital (SRRH) in Mwanza, Tanzania. SRRH is the University Teaching Hospital of the Catholic University of Health and Allied Sciences with a bed capacity of 315 and a total of 368 employees (SRRH – web link: http://mwanzarrh.go.tz/background). The SRRH Clinical Microbiology Laboratory processes an average of 90–100 paediatric blood culture samples per month.

### Study population, inclusion criteria and exclusion criteria

The study included all children aged 0–12 years with clinical signs and symptoms suggestive of BSI, for whom clinicians requested blood samples for culture and susceptibility testing. Participants were selected based on one or more criteria outlined in the sepsis screening tool from the WHO Young Infant Study Group [10]. These criteria included fever (above 38 °C or below 36 °C), age-specific tachycardia (heart rate >90 bpm), age-specific tachypnoea (respiratory rate >20 breaths per minute), convulsions, lethargy, cold extremities, frequent vomiting or reduced urine output. Children were excluded if they could not provide blood samples or if the sample volumes were insufficient to meet protocol requirements.

### Sample size calculation

The Kish formula (*n*=*z*² *p* (1 *p*)/*d*²) was used to determine the sample size [11], where *n* is the required sample size, *z* is the *Z*-score for 95% confidence interval (CI=1.96), *P* is the prevalence and *d* is the tolerable error(=5%). The previous prevalence of BSIs among children in Mwanza, Tanzania of 14.2% was used [8]. This resulted in a minimum of 188 children to be included. Considering a design effect of 1.6, a total of 302 children were enrolled in this study.

### Sampling technique and data collection

A serial sampling technique of participants who met the inclusion criteria was used to enrol the children until the required sample size was reached. Briefly, all blood culture samples, along with their completed request forms submitted at the SRRH microbiology laboratory, were reviewed to identify those with required inclusion criteria, followed by data collection from the enrolled children after obtaining the consent from the guardians/parents.

Demographic characteristics, including age, sex, address and ward of admission, were extracted from patient files and/or request forms. Clinical features indicative of BSIs or sepsis, such as fever (greater than 38 °C or less than 36 °C), elevated heart rate (over 90 bpm) and increased respiratory rate (over 20 breaths per minute), were retrieved from the patient’s clinical case notes, request forms or files.

### Sample collection and transportation procedure

Blood culture samples were collected by phlebotomists or doctors/paediatricians as previously described [12] while observing all required aseptic techniques [13]. About 2–5 ml of blood were aseptically collected based on the patient’s age to ensure a 1 : 10 ratio of blood to broth brain heart infusion (BHI) from Oxoid Ltd., UK [9,14], as presented in Table 1. The bottles containing the mixture of blood and BHI broth were transported to the laboratory within 2 h of collection and incubated at 35–37 °C [15].

**Table 1.** Age-based criteria, required volume of blood and respective broth medium

Age	Category	Vol. of blood	Vol. of BHI	Blood–broth ratio
Below 1 month	Neonates/infants	1.5–2 ml	15–20 ml	1:10
1–48 months	Infants/children under 5	2–2.5 ml	20–25 ml	1:10
5–12 years	Older children	3–3.5 ml	30–35 ml	1:10

Childrennder 5 are children below five years of age.

In this study, only one bottle of blood sample was collected per paediatric patient, as supported by several studies that had consistently concluded that a sufficient blood volume in a single blood culture bottle was adequate to detect pathogens [16,17].

### Laboratory procedures

**Fig. 1.**
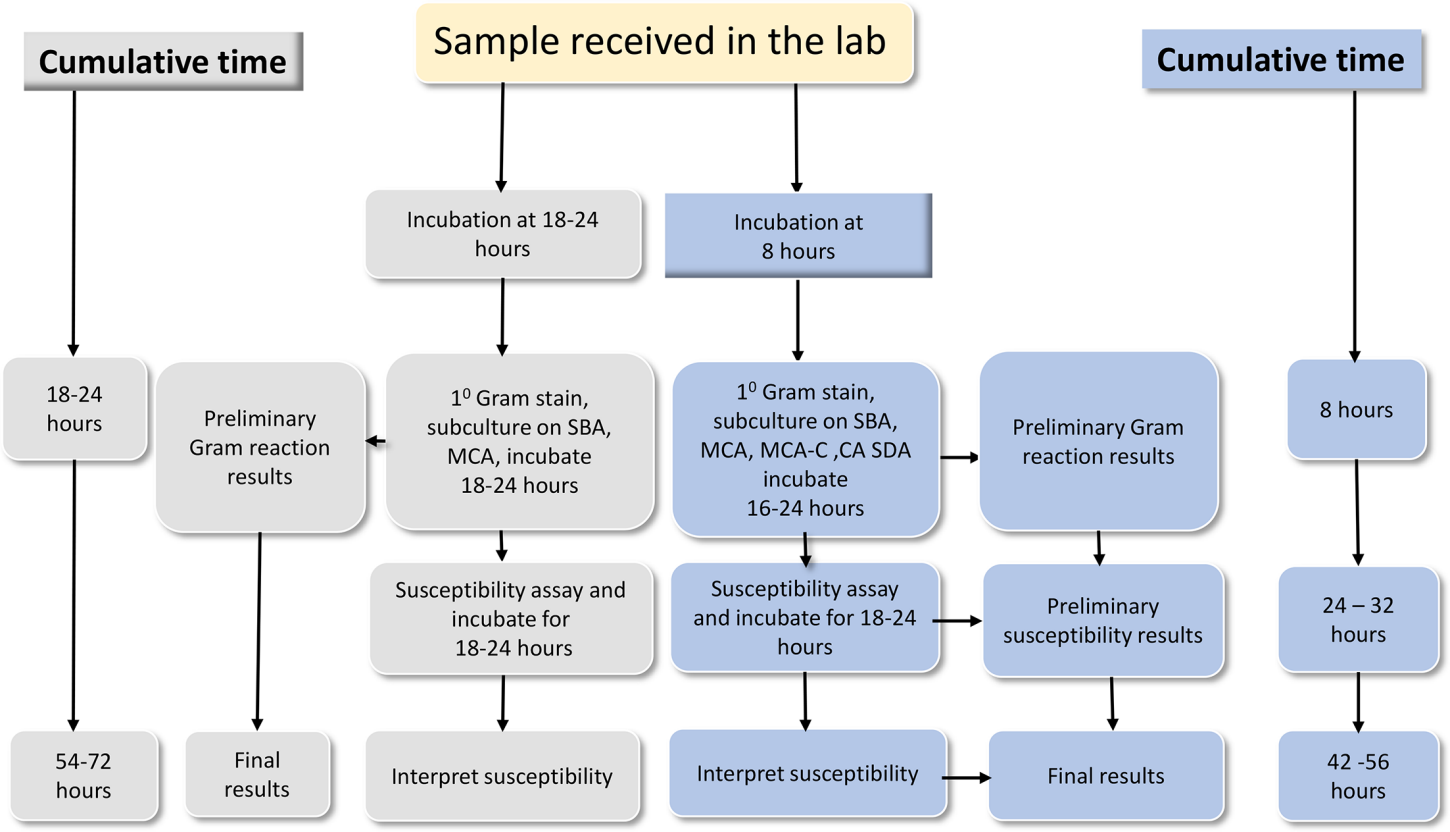
Current practices of blood culture at SRRH against the study protocol.

The standard manual (conventional) aerobic blood culture testing method was implemented in this study [18]. After 8 h of initial incubation of the blood culture sample [blood–broth (BHI) mixture in universal bottle] at 35–37 °C, a primary Gram stain was performed from the blood–broth (BHI) mixture followed by blind subculture into in-house prepared culture media; 5% sheep blood agar, chocolate agar, sabouraud dextrose agar and MacConkey agar with and without cefotaxime 2 µg ml^−1^ (Oxoid Ltd., UK). The blood culture sample was further incubated at 35–37 °C and blind sub-cultured at 24 and 120 h of incubation, as shown in Fig. 1.

### Identification of bacteria causing BSIs

Identification of bacteria involved plate readings of inoculated culture plates after 18–24 h of incubation at 35–37 °C and secondary Gram staining from pure colony growth on culture media. This was followed by the conventional physiological and biochemical methods of bacteria identification as previously described [19]. For Gram-positive bacteria (GPB), identification involved assessment of colony morphology and haemolytic activity on 5% sheep blood agar plates (Oxoid Ltd., UK), catalase and coagulase reactions. Other tests included the bile aesculin test and the standard antibiotic discs like bacitracin and optochin discs (Oxoid Ltd., UK) [18,19]. For GNB, identification included assessing colony morphology on 5% sheep blood and MacConkey agar, triple sugar iron agar reactions, hydrogen sulphide production, indole production, motility, Christensen’s urease activity, Simmons’ citrate utilization and Oxidase test [19].

### Antimicrobial susceptibility testing

The antimicrobial susceptibility of all isolates was determined using the Kirby-Bauer disc diffusion method on Mueller Hinton agar (Oxoid, UK) [20], following the Clinical and Laboratory Standards Institute guidelines [21]. The antibiotic discs for GPB included ampicillin (10 µg), clindamycin (2 µg), erythromycin (15 µg), ciprofloxacin (5 µg) and cefoxitin (30 µg) (Oxoid, UK). For GNB, the antibiotic discs included ampicillin (10 µg), amoxicillin/clavulanate (20/10 µg), ciprofloxacin (5 µg), tetracycline (30 µg), gentamicin (10 µg), trimethoprim/sulfamethoxazole (SXT) (1.25/23.75 µg), ceftriaxone (30 µg), ceftazidime (30 µg), cefepime (30 µg) and meropenem (10 µg) (Oxoid, UK).

ESBL production was detected using the disc approximation method, as previously described [22], while methicillin-resistant *Staphylococcus aureus* (MRSA) was confirmed using the cefoxitin disc (30 µg) [21]. Multidrug-resistant bacteria was defined as those that have become resistant to at least one agent in three or more classes of antimicrobial agents [23].

American Type Culture Collection (ATCC), including *E. coli* ATCC 25922, *S. aureus* ATCC 25923 and *Pseudomonas aeruginosa* ATCC 27853, was used for quality control of culture media, discs and incubation conditions [21].

### Data management summary

The study involved the initial data entry into a standard data extraction sheet, followed by transferring to a Microsoft Excel spreadsheet for comprehensive data cleaning. Descriptive analysis was carried out using STATA software version 15 (College Station, TX, USA), following study objectives. Analysis involved calculating the proportions of positive blood culture samples at 8, 24 and 120 h of incubation; the denominator was the total number of blood culture samples. The two-sample proportion test was used to compare the proportion of positive blood culture across these different incubation times. A 95% CI was calculated, and a *P*-value of 0.05 or less was considered statistically significant.

### Ethical considerations

This study was cleared by the joint Catholic University of Health and Allied Sciences/BMC Research Ethics and Review Committee with ethical clearance certificate number CREC/786/2024. Permission to conduct the study at SRRH in Mwanza, Tanzania, was sought and obtained from the Mwanza Regional Administrative Secretary. Informed consent was obtained from parents or guardians.

## Results

### Socio-demographic and clinical characteristics of study participants

The study enrolled 302 children; more than half were males (160, 53.0%). The median age of the children was 6 years (IQR: 1–7 years), and the most common age group was 6–12 years, representing 44.7% (143) of the children. The median duration (IQR) of hospital stay of the children at the time of enrolment was 2 days (IQR: 1–2 days). Fever was the most commonly reported clinical sign for 259 (85.8%) children. The median body temperature was 39 °C (IQR: 38–39 °C) (Table 2).

**Table 2.** Socio-demographic and clinical characteristics of patients enrolled

Patient characteristic	Category	No. (%)
Sex	Male	160 (53.0)
	Female	142 (47.0)
Age group	<1 month	25 (8.28)
	1 month to 5 years	135 (44.7)
	6–12 years	142 (47.02)
Presence fever	Yes	259 (85.76)
	No	43 (14.24)
Body temperature	>38 °C	243 (93.8)
	<36 °C	16 (6.2)
Heart rate	>90 min^−1^	125 (41.4)
	<90 min^−1^	177 (58.6)
Respiratory rate	>20 min^−1^	272 (90.1)
	<20 min^−1^	30 (9.9)
Use of antibiotics before clinical sampling (blood culture)	Yes	27 (8.9)
	No	275 (91.1)
Use of antibiotics in the past 2 weeks	Yes	15 (5.0)
	No	287 (95.0)
Duration of admission at the time of clinical sampling (blood culture)	0 days (outpatient)	93 (30.8)
	1 day	129 (42.7)
	>1 day	80 (26.49)
Presence of invasive devices	Yes	80 (26.5)
	No	222 (73.5)

### Proportion of pathogens isolated at 8, 24 and 120 hours of incubation

Microbiologically confirmed BSIs were detected in 90 (29.8%) children. The total incremental bacterial culture positivity detection was 46 (15.2%; 95% CI: 11.4–19.4), 77 (25.5%; 95% CI: 20.7–30.8) and 90 (29.8%; 95% CI: 24.7–35.3) at 8, 24 and 120 h post-incubation, respectively. Slightly more than half of the children with microbiologically confirmed BSIs were detected after blind subculture within 8 h of initial incubation, 51.1% (46, *n*=90), while an additional 31 (34.4%) and 13 (14.4%) children were detected after blind subculture within 24 and 120 h of incubation, respectively. The difference in blood culture positivity between 8 h (15.2%; 95% CI: 11.1–19.2) and 24 h (25.5%; 95% CI: 20.5–30.4) among 302 samples was statistically significant (two-sample test of proportions *P*=0.007).

### Patterns of pathogens isolated at 8, 24 and 120 h incubation

**Fig. 2.**
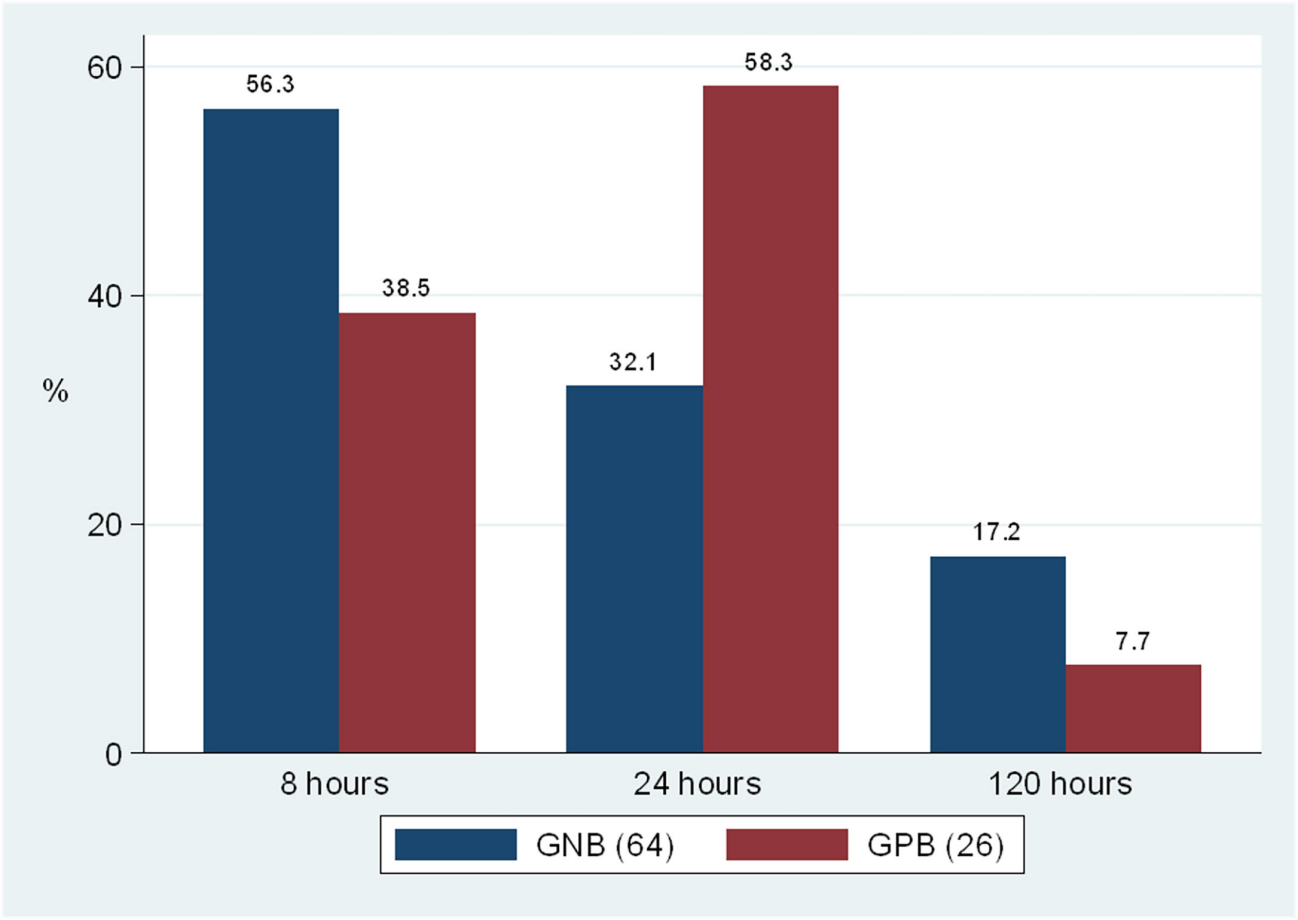
Proportion of bacteria isolated on subculture after 8, 24 and 120 hours of initial incubation based on Gram reaction.

Out of the 90 pathogens isolated after 120 h of blood culture incubation, 64 (71.1%) were GNB, while 26 (28.9%) were GPB. At 8 h post-incubation, GNB were detected in 36 (56.3%, n=64) of the children with Gram-negative BSIs, while GPB were detected in 10 (38.5%, n=26) of the children with Gram-positive BSIs. The blood culture positivity after 24 h revealed an increase in the yield by 17 (32.1%) and 14 (58.3%) isolates of GNB and GPB, respectively. After 120 h of initial incubation, there was an increase in the yield by 11 (17.2%) and 2 (7.7%) isolates of GNB and GPB, respectively (Fig. 2). The overall difference in the positivity at 120 h was not statistically significant compared with 24 h positivity (25.2 vs. 29.8%, *P*=0.3077).

The most frequently isolated bacterial pathogens were *K. pneumoniae* 23 (25.6%) and *S. aureus* 22 (24.4%). Notably, 14 (60.9%) of all *K. pneumoniae* isolates were detected after 8 h of incubation, with an additional 2 (8.7%) detected after 24 h of incubation. While 10 (45.5%) of *S. aureus* were detected after 8 h of incubation, significant addition of 12 (54.5%) were detected after 24 h of incubation (Table 3). All *Pseudomonas spp*. and *E. coli* were detected within 8 h of incubation. However, it is noteworthy that 11 GNB, consisting of 7 *K. pneumoniae*, 2 *Enterobacter cloacae*, 1 *Acinetobacter spp*. and 1 *Citrobacter freundii* isolate, exhibited delayed growth, requiring up to 120 h of incubation despite the typically short generation times of these bacteria (Table 3).

**Table 3.** Patterns of pathogens isolated at 8, 24 (new, undetected at 8 h) and 120 h (new, undetected at 8 and 24 h)

Pathogen	8** h**	24** h**	120** h**	Overall pathogen
*K. pneumoniae*	14 (60.9%)	2 (8.7%)	7 (30.4%)	23
*S. aureus*	10 (45.5%)	12 (54.5%)	0 (0%)	22
*Acinetobacter spp*.	9 (60%)	5 (33.3%)	1 (6.7%)	15
*E.cloacae*	2 (20%)	6 (60%)	2 (20%)	10
*C. freundii*	4 (44.4%)	4 (44.4%)	1 (11.1)	9
*Pseudomonas spp*.	4 (100%)	0 (0%)	0 (0%)	4
*E. coli*	3 (100%)	0 (0%)	0 (0%)	3
*Enterococcus faecalis*	0 (0%)	1 (33.3%)	2 (66.7)	3
*Streptococcus pneumoniae*	0 (0%)	1 (100%)	0 (0%)	1
Total	46 (51.1%)	31 (34.4%)	13 (14.4%)	90

### Antibiotic susceptibility patterns of pathogens causing BSI in children

The majority of GNB isolated after 120 h of incubation were sensitive to gentamicin (54.7%, 35/64) and ciprofloxacin (50%, 32/64). GNB resistance was highest to ampicillin (90.9%, 20/22), amoxicillin/clavulanate (82.2%, 37/45) and ceftriaxone (64.1%, 41/64). Notably, 40 (62.5%) of *K. pneumoniae* and other GNB were resistant to 3GC. Of the 36 (56.3%) GNB detected at 8 h of incubation, 20 (55.5%) exhibited growth on MCA-C, which indicates resistance to 3GC, with an additional 15 (37.5%) detected at 24 h of incubation. The ESBL phenotype was identified in 15 (65.2%) of *K. pneumoniae* isolated and 2 (66.7%) of *E. coli* isolated. The proportion of carbapenem resistance among GNB was 21.9%. Among the antibiotics tested against GPB isolated, the proportion of GPB resistance was highest to ampicillin (65.4%) and erythromycin (52.2%). Of the 22 *S. aureus* isolates, 10 (45.5%) were identified as MRSA (Table 4).

**Table 4.** AMR patterns of bacteria causing BSI in children

Antibiotic	*K. pneumoniae* (23)	*Acinetobacter* spp*.* (15)	*E. cloacae* (10)	*C. freundii* (9)	Other GNB* (7)	*S. aureus* (22)	Other GPB† (4)
Ampicillin	na	na	90.0%	100%	66.7%	68.15%	50.0%
Gentamicin	39.1%	20.0%	50.0%	55.6%	28.6%	na	na
Ciprofloxacin	39.1%	20.0%	30.0%	55.6%	28.6%	45.5%	0.0%
Tetracycline	52.2%	46.7%	20.0%	66.7%	57.1%	na	na
AMC	82.6%	na	70.0%	100%	66.7%	na	na
SXT	66.9%	33.3%	50.0%	77.8%	71.4%	na	na
Ceftriaxone	62.2%	73.3%	40.0%	66.7%	71.4%	na	na
Ceftazidime	52.2%	53.3%	40.0%	66.7%	28.6%	na	na
Cefepime	56.5%	40.0%	40.0%	66.7%	28.6%	na	na
Meropenem	13.0%	20.0%	30.0%	44.4%	14.3%	na	na
Erythromycin	na	na	na	na	na	54.6%	0.0%
Clindamycin	na	na	na	na	na	13.6%	25%

* *E. coli* (3) and *P. aeruginosa* (4).

† *E. faecalis* (3) and *S. pneumoniae* (1).

AMC, amoxicillin/clavulanic acid.

## Discussion

The prolonged turnaround time of manual blood culture tests, exacerbated by the lengthy initial incubation period (18–24 h), significantly hinders timely antibiotic treatment and increases mortality among children with BSIs. Early detection and initiation of appropriate antimicrobials is crucial in saving the lives of children with BSI. This study was conducted to determine the impact of reducing initial incubation time on culture results and patterns of bacterial pathogens detected.

Consistent with earlier research [24], which found that half of bacterial pathogens were detected after 4–14 h (an average of 8 h) of initial incubation, the current study shows similar findings, with more than half of children with BSI being detected in subculture done after 8 h of initial incubation. This indicates that early (8 h) blind subculture led to timely and appropriate treatment for about half of the children with microbiologically confirmed BSIs. The blood culture detection results after 24 h of initial incubation aligned with previous study findings that reported the detection of ~85% of microbiologically confirmed BSIs [25].

Consistent with the current study’s finding, studies conducted in similar settings from 2010 to 2020 reported that GNB are the predominant pathogens isolated from paediatric blood cultures (61.4–85.5%) [4,6, 7]. This indicates that the majority of children with BSIs are due to GNB. However, in this study, more than half of all GNB that were detected after 120 h of incubation were already detected at 8 h of initial incubation. This means that more than half of children with Gram-negative BSIs could receive appropriate treatment within 24 h.

The proportion of 3GC resistance among GNB in this study (62.5%) is higher than the 50% reported 13 years ago [9]. This could reflect the fact that AMR has been increasing over time. The overuse of 3GCs, especially ceftriaxone, contributes significantly to resistance due to adaptive mechanisms that cause cross-resistance within the antibiotic classes [6]. In contrast to our findings, studies conducted in similar settings 3 years ago reported much higher resistance to 3GC among GNB, with the rates of 74.7 and 93.2% [8,26]. The differences may be due to variations in healthcare settings, as it is well established that children admitted to tertiary hospitals are more likely to develop 3GC-resistant BSIs compared with those in regional hospitals, such as SRRH [8]. The WHO classifies *Enterobacterales* such as *Klebsiella* species resistant to 3GCs as critical priority pathogens list [27]. This highlights the urgent need for improved culture methods to enable earlier detection of 3GC-resistant GNB. In this study, half of the children with 3GC-resistant BSIs were detected at 8 h of initial incubation, allowing the initiation of appropriate treatment within 24 h. This is a significant improvement over traditional practices of sub-culturing after 24 h of initial incubation. The 8 h subculture technique, combined with blind subculture into MCA-C, allows faster detection, identification and susceptibility testing, enabling early detection of the GNB resistant to 3GC. This facilitates the prompt and appropriate treatment of children with BSIs.

In line with other studies in similar settings [8,9], *K. pneumoniae* was the most frequently isolated species from paediatric blood cultures. *Klebsiella spp*. predominance in paediatric BSIs is attributed to its environmental resilience and siderophore-dependent iron acquisition, enabling it to thrive in serum, leading to BSIs development [6]. Moreover, the selective pressure exerted by antibiotics may further reinforce the predominance of this organism [9]. In this study, the majority of *K. pneumoniae* (60.9%) were detected at 8 h of incubation, indicating that three-fifths of children with BSIs due to *K. pneumoniae,* the leading cause of BSI in children, could be initiated on appropriate treatment within 24 h.

The majority of GNB were detected at 8 h of incubation compared to GPB*;* this could be due to the shorter generation time of GNB compared to GPB. This study revealed a low yield of *E. coli* (3.3%), compared with previous studies in the Mwanza region, mainly at a tertiary hospital (BMC), which reported *E. coli* to be the second commonest GNB causing BSI in children accounting for 12.6 and 14.8% of the isolates [8,9]. The variations across facilities in Tanzania underscore the need for further research on facility-specific pathogen distribution to improve infection prevention and control, due to the current lack of comprehensive data. Interestingly, all *E. coli* and *Pseudomonas spp*. were isolated at 8 h of incubation, likely due to their short generation time ranging from 20 to 60 min, suggesting that children with BSI due to these pathogens could receive correct treatment within 24 h. However, 11 GNB showed delayed growth, requiring up to 120 h of incubation (Table 3). This may be due to the fact that nearly two-thirds of these GNB with delayed growth up to 120 h were isolated from blood cultures of children who were already on antibiotic use before sample collection, while none of the cases with a high yield (over 60% detection) at 8 h involved prior antibiotic use. This emphasizes the importance of blood culture collection before antibiotic use because antibiotics reduce pathogen yield and decrease sensitivity in blood culture tests [17]. In the present study, the high AMR observed among GNB aligns with two previous studies in the Mwanza region: ampicillin (97 and 98%), amoxicillin/clavulanate (88 and 90%) and ceftriaxone (51 and 75%) [6,7]. This poses a challenge in the management of sepsis among children because these antibiotics (ampicillin and ceftriaxone) are part of the treatment options in Tanzania, whereby ampicillin, cloxacillin and gentamycin are the first-line treatment, while ceftriaxone and gentamycin are the second-line treatment for children with sepsis [28].

Similar to previous studies [4,6, 7], a significant proportion of *K. pneumoniae* (65.2%) and *E. coli* (66.7%) isolates were ESBL producers, likely due to over use of 3GC like ceftriaxone in the study settings. The proportion of MRSA among *S. aureus* strains in this study was notably higher at 45.5%, in contrast to the 28 and 34.7% reported in Mwanza 13 and 4 years ago, respectively [8,9]. The increasing trend of MRSA among *S. aureus* strains indicates a growing challenge in managing GPB BSI in children. The overall increasing trend of AMR among GPB and GNB highlights the urgent need for strengthened infection control measures, enhanced antimicrobial stewardship and routine culture and antimicrobial susceptibility testing to guide effective treatment decisions at SRRH [8]. Furthermore, this suggests the need for including SRRH in the ongoing nationwide AMR surveillance, as outlined in the National Action Plan on Antimicrobial Resistance (NAP-AMR) 2023–2028.

### Study limitations

The study did not include anaerobic blood cultures, potentially omitting anaerobic bacteria. Although less prevalent, their exclusion could still affect the study’s findings. Another limitation was the inclusion of patients who had received antibiotics before blood culture collection, possibly leading to delayed pathogen growth and low yield. Moreover, some participants could not recall their medication history, though we cross-checked medical records and consulted caregivers where possible.

## Conclusion

Blind subculture after 8 h of initial incubation correctly detected more than half of the children with microbiologically confirmed BSIs. Incorporating blind subculture on MCA-C after 8 h of incubation resulted in the correct treatment of half of the children with BSIs caused by GNB within 24 h. A blind subculture within 8 h of initial incubation to reduce the turnaround time for blood culture results with incorporation of MCA-C for appropriate treatment within 24 h should be implemented.

## Abbreviations

AMR: antimicrobial resistance
ATCC: American Type Culture Collection
BHI: brain heart infusion
BMC: Bugando Medical Centre
bmp: beats per minute
BSIs: bloodstream infections
ESBL: exhibiting extended-spectrum beta-lactamase
3GCs: third-generation cephalosporins
GNB: Gram-negative bacteria
GPB: Gram-positive bacteria
IQR: interquartile range
MCA-C: MacConkey agar supplemented with cefotaxime 2 µg ml^−1^
MRSA: methicillin-resistant *Staphylococcus aureus*
SRRH: Sekou Toure Regional Referral Hospital
SXT: sulfamethoxazole
WHO: World Health Organization
